# Supplementary material for: Assessment of potential risk factors for COVID-19 among health care workers in a health care setting in Delhi, India -a cohort study
Source: PLoS One. 2023 Jan 20;18(1):e0265290. doi: 10.1371/journal.pone.0265290 (PMC9858779; doi:10.1371/journal.pone.0265290)
Supplement: S1 File — (DOCX) [file pone.0265290.s001.docx]

### S1 File: Sample size calculation, operational Definition, participant recruitment and sample processing

**Sample Size:**

Based on extensive review of literature (8,17), in Indian settings, we estimated the proportion of seroconversion in the general population (Po) at 25% and the proportion of seroconversion in health care workers (Pa) at 36%. Taking significance at 5% and power at 80, and putting them in two-sample proportionality hypothesis formula (*check supplementary material*), we calculated a sample size of 130, which was adjusted considering the attrition rate of 25% to 170.

$$n=\frac{\{Z_{1-\frac{\alpha}{2}} \sqrt{{P_{o}(1-P}_{o}})+Z_{1-\beta}\sqrt{{P_{a}\left( 1-P_{a} \right)}}{\}}^{2}}{{{(P}_{a}-P_{o})}^{2}}$$

Were

P_o_ = Population Proportion

P_a_ = Sample Proportion

α = Significant level

1-$\beta$ = Power

**Operational Definition**

- Type of exposure
  - Close contact exposure (within 1 meter)
    - Prolonged face-to-face exposure (> 15 minutes)
    - Exposure during aerosolizing procedures
    - Direct Exposure with body fluid
  - Patient’s materials exposure: (personal belongings, linen and medical equipment)
    - Exposure to Patient’s body fluid via materials exposure
  - Surface exposure:
    - Exposure to Patient’s body fluid via surface around the patient
- Category of Healthcare worker:
  - Category I – All doctors, including faculties, residents, and medical interns.
  - Category II – Nurses and nursing assistants
  - Category III – Lab assistants, technicians, field workers, housekeeping, sanitation workers, security personnel, general duty attendants, pharmacist, reception,
- Category of Workplace:
  - High risk area: areas where the risk of COVID-19 infection was high such as the emergency, aerosol generating procedure rooms, COVID-19 wards, Intensive Care (ICU), labor room and testing center.
  - Low risk area: Areas where there is a low risk of COVID-19 infection such as the General Outpatient department, offices, laboratories, reception, inquiry counter and security posts.

**Participant Recruitment (Supplementary Figure 1)**

*Baseline assessment:*

All HCW recruited into the study completed a researcher-administered, translated questionnaire at baseline, which covered:

- socio-demographic information
- training on infection prevention and control measures
- adherence to infection prevention and control measures
- type of exposure to the COVID-19-infected
- status of vaccination
- a baseline serum sample for assessing seropositivity.

*Follow-up and Endline assessment:*

The participants were given a symptoms diary for documenting symptoms of COVID-19 during the 21 days follow-up period. They were contacted to assess their symptomatology and reminded to fill symptom diary. We did the end-line visit at 22-28 days from the first visit, during which end-line questionnaire and second serum sample were collected. The serum samples were tested for antibodies against COVID-19 using the WANTAI serological testing kit. The paired serological samples (at baseline and end-line) was utilized for calculating the seroconversion rate, a proxy for the secondary infection attack rate.

**Sample collection and processing:**

Two milliliters of blood were collected twice by venepuncture from all HCWs enrolled in the study. All the blood sample was coded with the participant unique ID and anonymized so that laboratory staff does not have access to the participant’s information. The first sample collected after enrolment was considered the baseline blood sample. All the subjects were recalled after 21 days (from the date of baseline sample collection) for the collection of endline blood sample. (*Protocal deviation 1*: In the initial proposal, we planned to call only those sero-negative at baseline; later, after protocol deviation, all HCW were called for endline). The paired samples testing protocol helped detect asymptomatic carriers and understand the pattern of seroconversion.

The sample was collected in the appropriate vial, coded, and allowed to stand upright for 30 minutes at room temperature, followed by centrifugation at 2500 rpm for 5 minutes before processing for testing the Anti-SARS-CoV-2 antibody. The blood collection staffs were well trained in safe specimen handling practices and spill decontamination protocols and used appropriate Personal protective equipment (PPE) during the sample collection process.

*Anti-SARS-CoV-2-total antibody detection:*

In this study, we employed the Wantai SARS-CoV-2-Ab ELISA kit, which detects total antibodies against the SARS-CoV-2 virus and is based on the principle of two-step incubation antigen “sandwich” enzyme immunoassay. Briefly, 100 ml of the patient’s serum is added to polystyrene microwell strips pre-coated with recombinant SARS-CoV-2 antigen. Three wells are marked, one well as a negative calibrator and two wells as a positive calibrator. A 50 ml negative and positive calibrator were added to respective wells, and the plate was incubated at 37^0^C for 30 minutes. Post incubation, the wells were washed five times with diluted wash buffer. 100μl of HRP-Conjugate was added to each well and incubated at 37^0^C for 30 minutes. The wells were rewashed five times, and then 50μl of Chromogen Solution A followed by 50μl of Chromogen Solution B was added into each well. The plate was then incubated at 37°C for 15 minutes in the dark. 50μl of Stop Solution was added into each well and mixed gently. Absorbance was measured using PR4100 microplate reader, Bio-Rad, USA (dual filter) with reference wavelength at 600~650nm. The cut-off value (C.O.) was calculated as C. O= Nc + 0.16 (Nc = the mean absorbance value for three negative calibrators). The tested serum samples were stored at -80^0^C with proper labelling.

*Test result Reporting:*

Those samples which had absorbance (A) less than the Cut-off value (C.O.), were reported as negative results, meaning no SARS-CoV-2 antibodies were detected with WANTAI SARS-CoV-2 Ab ELISA (A / C.O. < 1). Specimens with absorbance equal to or greater than the Cut-off value were considered positive. After decoding, the study participants were informed about their baseline and endline antibody results, and counselling was done accordingly.
