# Supplementary figures and images for: Assessment of potential risk factors for COVID-19 among health care workers in a health care setting in Delhi, India -a cohort study
Source: PLoS One. 2023 Jan 20;18(1):e0265290. doi: 10.1371/journal.pone.0265290 (PMC9858779; doi:10.1371/journal.pone.0265290)

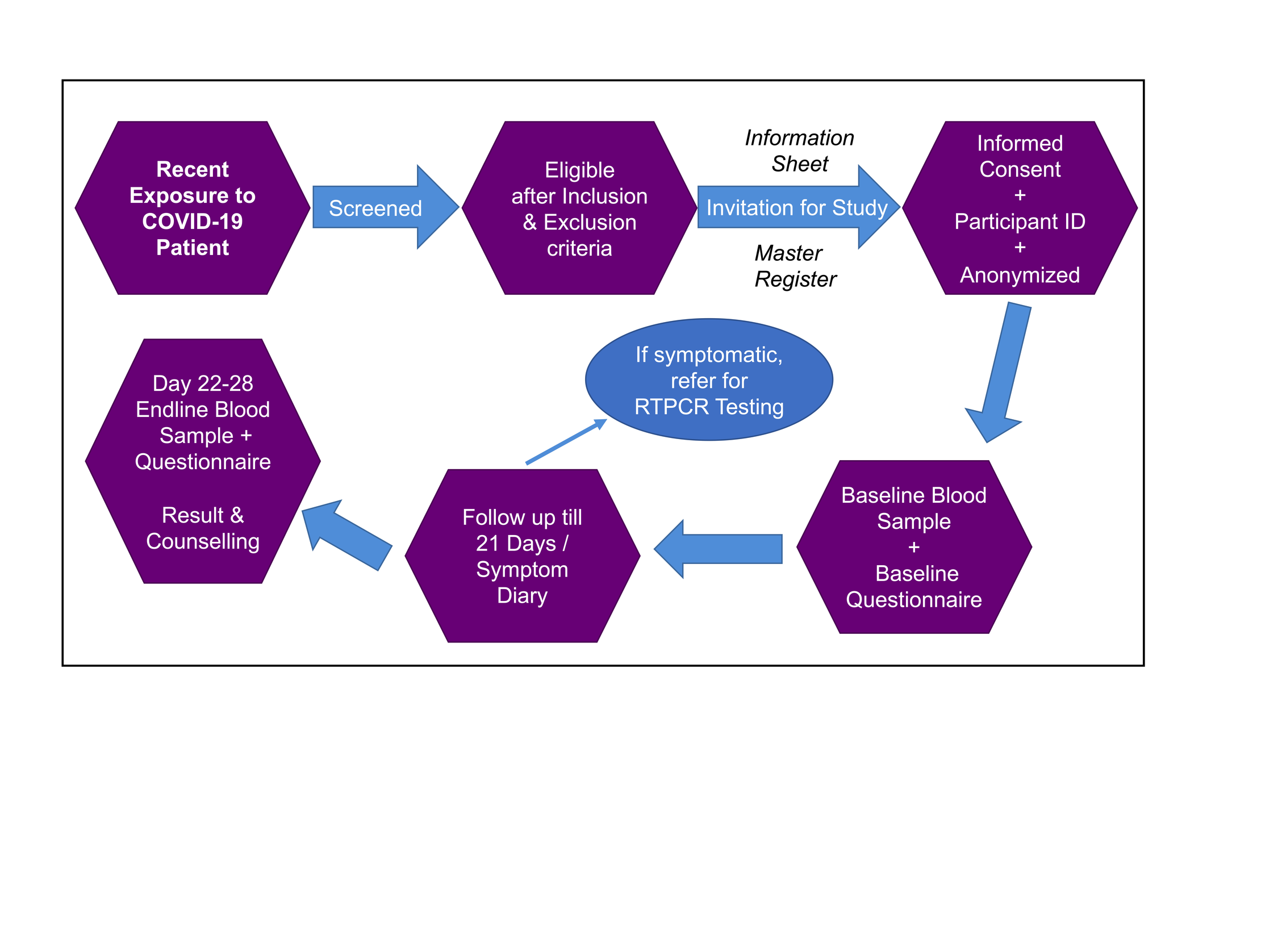

Supplement: S1 Fig — (TIF) [file pone.0265290.s002.tif]

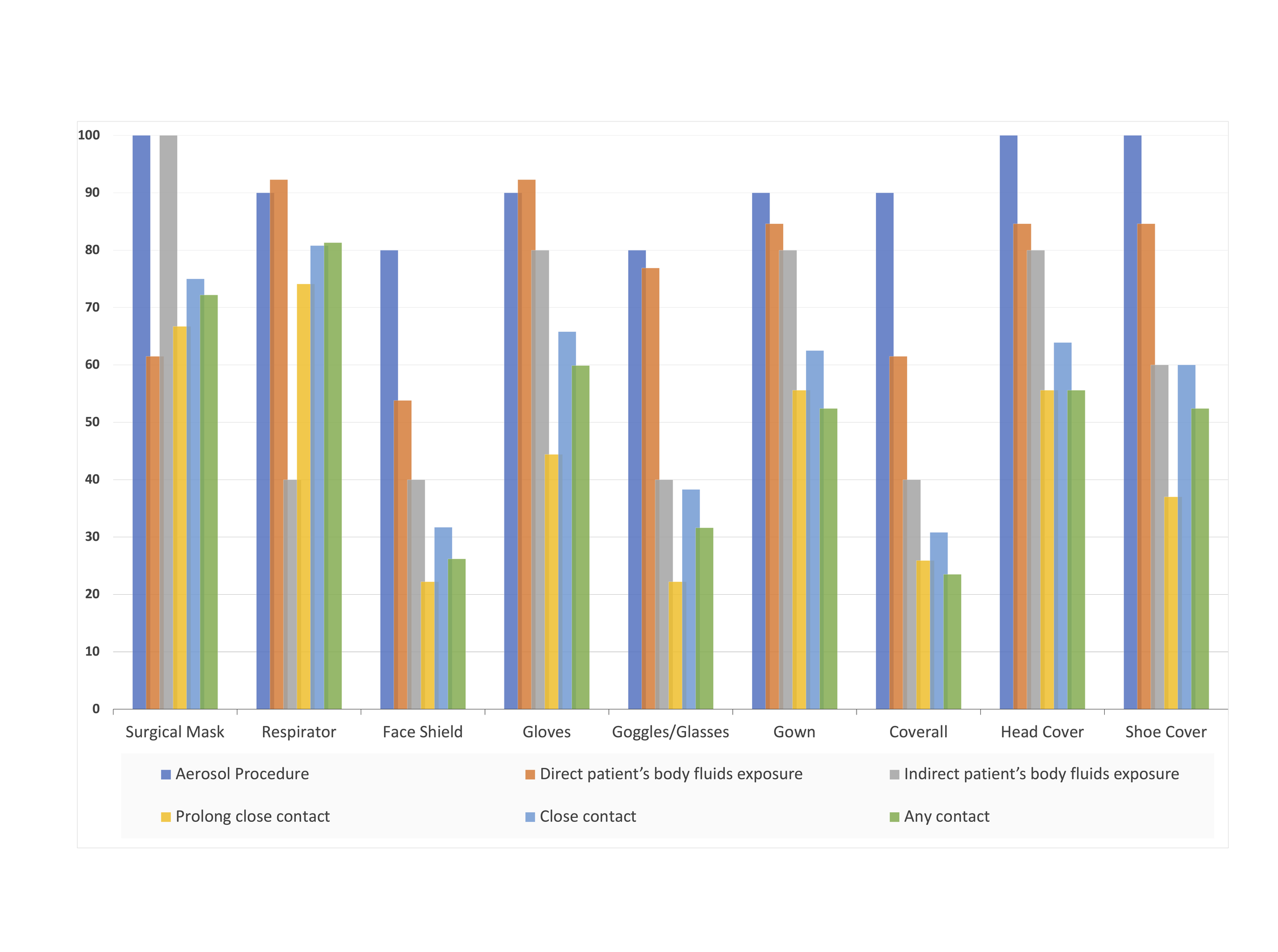

Supplement: S2 Fig — (TIF) [file pone.0265290.s003.tif]
