# Supplementary material for: Assessment of potential risk factors for COVID-19 among health care workers in a health care setting in Delhi, India -a cohort study
Source: PLoS One. 2023 Jan 20;18(1):e0265290. doi: 10.1371/journal.pone.0265290 (PMC9858779; doi:10.1371/journal.pone.0265290)
Supplement: S1 Table — (DOCX) [file pone.0265290.s004.docx]

**S1 Table: Hand hygiene practiced by healthcare workers**

| **Hand Hygiene** | **Always As Recommended** | | | | **Most of the time** | **Occasionally** |
| --- | --- | --- | --- | --- | --- | --- |
|  | Overall | Doctors | Nurse | Paramedics | Overall | Overall |
| **Before touching patient** | 98.4% | 96.1% | 100.0% | 100.0% | 1.6% | 0.0% |
| **Before cleaning septic procedure** | 98.4% | 96.1% | 100.0% | 99.0 | 1.6% | 0.0% |
| **Body fluid exposure** | 100% | 100% | 100% | 100% | 0.0% | 0.0% |
| **After touching patient** | 98.4% | 94.1% | 100% | 100% | 0.5% | 1.0% |
| **After touching surrounding** | 96.4% | 86.3% | 100% | 100% | 2.6% | 1.0% |
| **Follow IPC standards** | 95.6% | 93.6% | 97.1% | 97.2% | 2.5% | 1.9% |
| **Wear PPE indicate** | 99.5% | 98.0% | 100% | 100% | 0.0% | 0.0% |

*Percentages may not total 100 because of rounding.
