# Supplementary material for: Assessment of potential risk factors for COVID-19 among health care workers in a health care setting in Delhi, India -a cohort study
Source: PLoS One. 2023 Jan 20;18(1):e0265290. doi: 10.1371/journal.pone.0265290 (PMC9858779; doi:10.1371/journal.pone.0265290)
Supplement: S2 Table — (DOCX) [file pone.0265290.s005.docx]

**S2 Table: Distribution of Symptom Profile with the serology at baseline (N=192)**

|  | **Baseline** | | **Follow up** | |
| --- | --- | --- | --- | --- |
| **Symptom** | Total n=192 | Seropositive n=119  (% among seropositive) | Total n=139 | Seropositive n=108  (% among seropositive) |
| **Any Symptom** | 11 (5.7%) | 3 (2.5%) | 14 (10.1%) | 5 (4.6%) |
| **Fever** | 0 (0%) | 0 (0%) | 2 (1.4%) | 1 (0.9) |
| **Sore throat** | 2 (1.0%) | 1 (0.8%) | 6 (4.3%) | 3 (2.8) |
| **Cough** | 2 (1.0%) | 1 (0.8%) | 4 (2.9%) | 2 (1.9) |
| **Shortness of breath** | 2 (1.0%) | 0 (0%) | 1 (0.7%) | 1 (0.9) |
| **Rhinitis** | 0 (0%) | 0 (0%) | 4 (2.9%) | 1 (0.9) |
| **Chills** | 1 (0.5%) | 1 (0.8%) | 0 (0%) | 0 (0%) |
| **Nausea** | 1 (0.5%) | 0 (0%) | 0 (0%) | 0 (0%) |
| **Vomiting** | 0 (0%) | 0 (0%) | 1 (0.7%) | 0 (0%) |
| **Headache** | 5 (2.6%) | 2 (1.7%) | 2 (1.4%) | 0 (0%) |
| **Rash** | 1 (0.5%) | 0 (0%) | 0 (0%) | 0 (0%) |
| **Muscle aches** | 1 (0.5%) | 0 (0%) | 2 (1.4%) | 1 (0.9) |
| **Joint ache** | 1 (0.5%) | 1 (0.8%) | 0 (0%) | 0 (0%) |
| **Loss of appetite** | 1 (0.5%) | 0 (0%) | 0 (0%) | 0 (0%) |
| **Loss of Sense/taste** | 1 (0.5%) | 0 (0%) | 0 (0%) | 0 (0%) |
| **Fatigue** | 2 (1.0%) | 2 (1.7%) | 4 (2.9%) | 1 (0.9) |
| **Other symptoms** | 1 (0.5%) | 1 (0.8%) | 0 (0%) | 0 (0%) |

*Percentages may not total 100 because of rounding.
