# Supplementary material for: Assessment of potential risk factors for COVID-19 among health care workers in a health care setting in Delhi, India -a cohort study
Source: PLoS One. 2023 Jan 20;18(1):e0265290. doi: 10.1371/journal.pone.0265290 (PMC9858779; doi:10.1371/journal.pone.0265290)
Supplement: Attachment — Submitted filename: final Reply of Reviewer 2 comments in detail point.docx [file pone.0265290.s007.docx]

**Reply of Reviewer 1 comments in detail point-by-point**

|  | Reviewer 1 comments | Authors reply |
| --- | --- | --- |
|  | 1. If the authors have adequately addressed your comments raised in a previous round of review and you feel that this manuscript is now acceptable for publication, you may indicate that here to bypass the “Comments to the Author” section, enter your conflict of interest statement in the “Confidential to Editor” section, and submit your "Accept" recommendation.  Reviewer #1: (No Response) |  |
|  | 2. Is the manuscript technically sound, and do the data support the conclusions?  Reviewer #1: Partly | We are grateful to the reviewer for providing valuable comments. We have taken comments seriously and thoroughly revised the relevant section. We request you to go to the revised manuscript which is in track mode in MS word. You can see all the changes done in the revised manuscript. |
|  | 3. Has the statistical analysis been performed appropriately and rigorously?  Reviewer #1 Yes | We are happy that reviewer now considered  statistical analysis done in the revised manuscript appropriate and rigorous. We hope all other lacunas as mentioned by them would be fulfilled in current version of manuscript. |
|  | 4. Have the authors made all data underlying the findings in their manuscript fully available?  Reviewer #1: Yes | We are grateful to you for pointing this out. |
|  | 5. Is the manuscript presented in an intelligible fashion and written in standard English?  Reviewer #1: yes | We are happy that reviewer now considered the revised manuscript presented in intelligible fashion. We have further improved the English as suggested by the reviewer. We are grateful to the reviewer for the overall improvement in the manuscript. |
| General comments: | The authors have addressed the comments sufficiently. Nevertheless, the manuscript needs some improvements prior to publication.  Despite the authors’ mention that a professional English reviewer/editor has reviewed the manuscript, some spelling mistakes are still observed in the manuscript, as well as conceptual errors (use of COVID-19 to refer to the virus, not for the disease), and readability still needs to be improved. Some examples:  - 1st paragraph introduction: “COVID-19 belongs to the large family of viruses Coronaviridae, the viruses have the peculiar property of constantly changing and becoming diverse, which has helped them spread and survive and jeopardize any health system.” It should read (for example): “SARS-CoV-2 virus belongs to the large family of viruses Coronaviridae. This virus has constantly been changing and becoming diverse, and this property has helped this virus to spread among vulnerable populations and jeopardize any health system quickly”. – like this edit, many parts of the manuscript could be improved | The authors have further improved the grammar and conceptual errors as suggested by the reviewer, to improve its readability, clarity and make it technical sound.  - Corrections done. Thankyou for pointing out the error |
|  | - 1st paragraph introduction: Coronaviridae needs to be in italics | - Correction done. Thankyou for |
|  | - 1st and second sentences in the 1st paragraph of the introduction: Use SARS-CoV-2 to refer to the virus and COVID-19 to refer to the disease (example: SARS-CoV-2 virus belongs... / SARS-CoV-2 transmits from... / the rest of the paragraph using COVID is correct). | - correction done. Thankyou for pointing out the error |
|  | The use of acronyms needs revision as well. - HCW appear for the first time in the second paragraph of the introduction, and the acronym is not explained (HCW providing COVID-19 care are at increased... - Should read as: “Healthcare workers (HCW) providing COVID-19 care are at increased...”; please check for consistency on other acronyms. | - correction done. Thankyou for pointing out the error |
|  | Also, some statistical analysis and data visualization in tables and figures need to be improved. P. ex.  You should check the use of Mean ± SD and Median (IQR), as it depends on the type of variable and distribution that one is better used than the other. Please review and modify the manuscript accordingly. Further details are provided below. | We are thankful to the reviewer for pointing out the error. We have corrected it in the current version of manuscript. |
|  | Please review the acronyms as there are still mistakes: for example: confidence intervals are written entirely although acronyms defined, etc. Some acronyms are defined but only used once (HICC, HAIs, HAHC, RPAC). Please check carefully. | - correction done. Thankyou for suggestion |
|  | References still need to be reviewed; please consider ENE COVID Study or other HCW seroprevalences studies in Spain, for example. | Thankyou for suggestion. The ENE COVID study is a population-based sero-epidemiological study from Spain.  In our manuscript we have tried to included studies from different countries done among HCW and one study at Spanish hospitals is included (Garcia-Basteiro et al). |
| Specific comments: |  |  |
| Introduction | 1. “India has the largest number of confirmed cases in Asia and the second-highest number of confirmed cases in the world” – state the period: the whole pandemic? 1st wave? 2nd? 1st and 2nd? | This statement is for whole pandemic. For clarity, we have added ‘till date’ in the sentence. |
|  | 2. Regarding the study objectives, the way they are written in the introduction does not sound adequate for a scientific article “So, the key research question we want to explore in this study is: Does providing COVID care make HCW vulnerable to COVID-19 infection compared to the general population? If yes, what are its risk factors? We did this study with the objectives (1) to understand the extent of human-to-human transmission and to find out risk factors for COVID19 among HCW in recent contact with a COVID-19 patient; and (2) to evaluate the effectiveness of infection prevention and control (IPC) measures among HCW.” I consider it should be reviewed and consider re-writing the whole last part of the last paragraph in the introduction as: “The serological data among Indian HCWs is limited. A countrywide serosurvey after the first wave observed seroprevalence among HCW at 25.6%, while it was 12.1% among HCW from teaching hospital in Delhi (14,15). Investigating the serological response and assessing the potential risk factors among health workers may help characterize virus transmission patterns, prevent future infections of health workers, and prevent the healthcare-associated spread of COVID-19. Thus, this study aims to understand the extent of human-to- human transmission and to find out risk factors for COVID-19 among Indian HCW and to evaluate the effectiveness of IPC measures among them.” – please note IPC acronym has already been defined in the previous paragraph | We are thankful to the reviewer for such an elaborated and useful suggestion. We agree with the reviewer that research question is not intuitive and has been removed in the current manuscript. We also have reframed the objectives as suggested. |
| Methods | Sample size: 1. Based on extensive. The article is missing p.ex: based on an extensive... 2. The formula can be in the supplementary material, although I consider that it is not needed. If excluded, please review the use of the acronyms in that paragraph. | We have added reference of the study based on which we estimated the sample sise. Also, the formula is moved to supplementary material, as suggested. |
|  | Participant recruitment: 1. When a confirmed case of COVID-19 was admitted or detected, all its suspected contact were traced by the Covid Surveillance Unit. –> contacts in plural? | We are thankful to the reviewer for pointing out the error. We have corrected it in the current version of manuscript. |
|  | 2. those eligible were invited and provided a Patient Information Sheet explaining the study. -> those eligible were invited to participate in the study and a Patient Information Sheet explaining the study was provided to all of them. | We are thankful to the reviewer for pointing out the error. We have corrected it in the current version of manuscript.  !! |
|  | Serology assessment: please check acronyms as Cut-off value (C.O.) is stated twice. | Acronyms are now properly used in the current manuscript. Thank you for pointing out. |
|  | Statistical analysis: 1. p-value less than 0.05 -> p-value <0.05 | We are thankful to the reviewer for pointing out the error. We have corrected it in the current version of manuscript. |
|  | 2. The categorical variables were presented as percentages (%) while mean/median and standard deviation/ interquartile range (IQR) were calculated for continuous variables à it must be clearer were mean and median are used as well as when SD or IQR; also, IQR acronym was defined previously, please check | We have revised the sentence adding clarity. IQR is used in Statistical analyses section first in the manuscript. |
|  | In the ethical considerations section, I still miss the compliance of any international guideline/code of practice/ legislation. This needs to be clarified. | Thankyou for pointing this out. We have followed Guidelines of International Conference on harmonisation – Good clinical practices and added a sentence in ethical considerations section. |
| Results | 1. (participation rate 192/395)--> consider % 2. Of them, 139 were also --> add % | Both percentages added as suggested |
|  | 3. The lowest percentage of unvaccinated individuals (121/192) was among doctors (47.1%), while paramedics had the lowest rate (9.8%) among fully vaccinated, and this difference was statistically significant (Figure 2).  à consider wording/modify (suggestion: “The lowest percentage of unvaccinated individuals was among doctors (N=121, 47.1%), while paramedics had the lowest rate (9.8%) of fully vaccinated individuals (p<0.001) (Figure 2).).  ...among them, 27 had prolonged face-to-face exposure, and 10 had exposure during the aerosol generating procedure, while exposure with body fluid was observed in 10.7%. -- use N or % or both N(%) or % (N/N); but be consistent. | Thankyou for the useful suggestions. We have revised the result section using N(%) consistently. |
|  | 4. We also assessed the risk of seropositivity, seroconversion, and rise in titre as highlighted in table 7. The seropositivity was significantly and negatively associated with doctor as profession [Odds Ratio (OR):0.35, 95% Confidence Interval (CI):0.18-0.71], COVID-19 symptoms [OR:0.21, CI:0.05-0.82], comorbidities [OR:0.14, CI: 0.03 - 0.67], and IPC training [OR:0.25, CI:0.07 - 0.86], while positively associated with middle age [OR 2.23, CI:1.19 -4.19], partially [OR:3.30, CI: 1.26-8.69], as well as fully vaccination for COVID-19 [OR:2.43, CI:1.12-5.27]. --> please be consistent with how results are written and exposed and review the use of previously defined acronyms. | We have used Odds Ratio (OR) and Confidence Interval (CI) acronyms in abstract before results section. As suggested, now we have removed them from results section. |
| Discussion: | 1. Please be consistent with how results (OR and CI) are written and review the use of previously defined acronyms.  Some parts of the discussion could be better developed, although the authors have tried to improve this section. The explanation for the results should be better performed in different parts of the discussion and also increase the comparison with previously published studies. | We are thankful to the reviewer for pointing this inconsistency. In the current manuscript we have revised as per recommendation. |
|  | 2. The way the discussion is started does not seem appropriate; the wording of the 1st paragraph should be reviewed. | Your suggestion have been incorporated in the revised manuscript and we have redraft the first paragraph of discussion section. |
| Limitations | 1. Because of lack of time, we had a high refusal rate to participate, especially by doctors and nurses, as they were exhausted in their covid duty, which could bring selection bias.  à the lack of time of who? Rewrite. Covid or pandemic duty?  2. We also had a higher attrition rate than expected, although the profile of responders and non-responders did not vary significantly. -> please refer to this information regarding the significance and please try to explain why this could be.  3. Confirmation with RT-PCR testing might have helped --> to what? It does not read clear. | Yes, the many HCW participants prospects did not gave consent stating lack of time after their COVID duties.  We have considered your feedback and reframed the limitation section to make it clear. Thankyou. |
| Conclusion & policy consideration | Conclusions could improve. 1. Consider maintain “conclusions" as heading, I think it is clearer. 2. We are in opinion that it would be appropriate to regularly test all healthcare workers for COVID-19, using both PCR and serological assays, irrespective of exposure or symptom history to protect this workforce. --> please re-write it is not technically sound enough. | We had used Conclusion & policy consideration as it was recommend in IJID guidelines. As suggested we have revised it now.  We have revised the conclusion section as per your suggestion. We are thankful for the same. |
| Tables and Figures | - COVID-19 denotes coronavirus disease 2019 --> does not need to be in the foot tables as it is widely used, and has not been explained this acronym in the manuscript  - Table 1: decide which is better to describe the “Incubation period”: Mean ± SD or Median (IQR). It does not make sense to use both. Please see previous comments regarding this. | Thank you for your suggestion. We have incorporated both of them in the revised manuscript. For describing Incubation period in our study have used Median and IQR as the data was not normal. |
|  | - Table 2: add significance (p-value) and a footnote explaining the test used for calculation; Direct Exposure -> exposure does not need capital letters.  - Table 3: column title can be p-value instead of significance; please be consistent in the different tables. A footnote must be added in the table to specify the test used for calculation.  - Table 4: A footnote needs to be added to specify the test used for calculation/ .010 à missing a “0” --> 0.010 / Prolong face to face à prolonged? /# Prolong contact was considered when it was >15min face to face --> prolonged?  - Table 5: Adq: adequate --> Adequate? / 3 (60%) 2 (40%) à consistency, always 1 decimal? Please check the whole manuscript / .759 - .188 --> consistency add 0.XXX? Please check the whole manuscript. | As suggested, we have added the significance for each variable. We have rewritten the footnote specify the test used and comments raised by reviewer.  We have used p value to three decimal places and percentage to one decimal placed in the table. |
|  | - Table 6 I would suggest not adding 95%CI but instead p-values comparing the different groups. Please, consider. Also, the 95%CI refers to the % and needs to be clarified if maintained. | We are in opinion that 95% CI of the variable is beneficial for the manuscript, thus have maintain. We have added clarification in footnote as suggested by the reviewer. |
|  | - Table 7: please be consistent and put the reference category always the first or the last to make it easy for the reader | Thank you for pointing this out. We have made last category of all the variable as reference category throughout the table. The authors have redrafted the whole manuscript. The final version that we are now submitting is after getting edited by professional editing service. |
