# Supplementary material for: Assessment of potential risk factors for COVID-19 among health care workers in a health care setting in Delhi, India -a cohort study
Source: PLoS One. 2023 Jan 20;18(1):e0265290. doi: 10.1371/journal.pone.0265290 (PMC9858779; doi:10.1371/journal.pone.0265290)
Supplement: Attachment — Submitted filename: final Reply of Reviewer comments.docx [file pone.0265290.s006.docx]

**Reply of Reviewer 1 comments in detail point-by-point**

|  | Reviewer 1 comments | Authors reply |
| --- | --- | --- |
|  | 1. Is the manuscript technically sound, and do the data support the conclusions?  Reviewer #1: Partly | We are grateful to the reviewer for providing valuable comments. We have taken this comment seriously and thoroughly revised the relevant section.  We request you to go to the revised manuscript which is in track mode in MS word. You can see all the changes done in the revised manuscript. |
|  | 2. Has the statistical analysis been performed appropriately and rigorously?  Reviewer #1: No | We have revised the results section and combined the figures and tables as suggested by the reviewer. We hope the current manuscripts fills the lacunae as stated by the reviewer. |
|  | 3. Have the authors made all data underlying the findings in their manuscript fully available?  Reviewer #1: Yes | We are grateful to you for pointing this out. |
|  | 4. Is the manuscript presented in an intelligible fashion and written in standard English?  Reviewer #1: No | We have redrafted the manuscript and thoroughly revised the result and discussion section. We have taken the help of an external agency for improving the written English. We believe that the current version of manuscript is an improvement over the previous one. |
|  | Comments regarding the use of English language:  There are some grammatical changes required to address the English language and style. Through the whole document, the English should be reviewed to increase the readability of the document and also to sound more technical. Spelling need to be checked carefully (COIVD 19 for example). Keywords should not be acronyms. Authors should spell the complete word/term instead. Acronyms are not appropriately defined and should be reviewed.  CI and OR should be reviewed to include 2 or 3 decimals consistently through the whole document. The manuscript should carefully be reviewed.  Please complete a thorough proof read of the text and correct any spelling and grammar errors. | The manuscript has been revied by a native English speaker for correcting language and style. We have taken the help of an external agency for improving the written English. We believe that the current version of manuscript has improved in readability, clarity and is technical sound.  Spelling and Acronyms have be checked and Keywords have been revised.  As suggested CI and OR are made consistent. |
|  | Title should be reconsidered. Please revise the title of your manuscript to include the research question, study design and setting. Regarding authors information, I miss information about Neetushree author as well as detailed information regarding the corresponding author.  One of the main weaknesses is the lack of updating of the bibliographic references. It is evident that the bibliography search should be reviewed and updated, because the manuscript has not considered many of the main seroprevalence studies in health workers. I would suggest performing an accurate review of similar papers already published on this topic. References should be updated.  Also, bibliographic references are often poorly cited. References should be carefully reviewed. | The title of the study is ‘Assessment of Potential Risk Factors for COVID-19 among Health Care Workers in a Health Care Setting in Delhi, India -A Cohort Study’ mentioned study design (Cohort Study) and setting (Health Care Workers in a Health Care Setting in Delhi, India). Since it is already long, we think that adding research question will make it non intuitive and decrease readability. Instead we have added the research question at the end of introduction section.  Mis-information about one author has been corrected, as pointed out.  With regards to the bibliographic references in introduction section, we have tried to build the rationale of our study and included those studies which were done in initial phase of pandemic, prior to designing our study. Although we have updated references in discussion section. |
|  |  |  |
| Specific comments: |  |  |
| Introduction | 1. I miss a reference at the end of the second paragraph. Introduction should be more concise and add what is already published regarding infection among HCW with more detail. | We are thankful to the reviewer for pointing out the omission of the reference. We have added it in the current version of manuscript.  As suggested, we have worked on our introduction section and added few articles to make it concise and relevant. |
|  | 2. The objective of the study is not accurate and should be better defined ant the end of the introduction section. | We have considered your feedback and reframed the objectives to make them accurate. Hope the new manuscript is able to fulfil the objectives of the study in a better way. |
| Material & methods: | 1. There is an incongruence of the study period in the abstract and the methods section. The abstract mentions 1st and 2nd wave while methods only mention second wave.  Methods should include information regarding the months in which first and second covid waves took place in the region. Please review. | Thank you for pointing out errors in abstract and the methods section. It has been corrected in the revised manuscript. |
|  | 2. Authors say “The study population included all the health personnel who were working in this hospital and had come in contact or been exposed recently to a COVID-19 patient receiving care” it should be stated clearly how many HCW and their working status and of whom, how many participated in the study. This information is not clear. The participants’ flowchart should be mentioned at this point. | We think that participant enrolment needs clarity in the study population. So we have added participants’ flowchart in the methods section. |
|  | 3. I do not really see the point of calculating sample size according to the design of the study... It will be better to assess the statistical power of the analysis according to the study population. | Since this was a Cohort Study, we observed and followed only those cases that had an exposure to COVID-19 patients. While designing the study, we didn’t plan to observe any unexposed groups.  For power analysis, we needed the data of exposed and unexposed group to test for development of antibodies against COVID-19, so we could not assess as the reviewer had suggested. But since there was some confusion regarding sample size in the previous version of manuscript, we have now calculated the sample size using population proportion formula to make it more clear. |
|  | 4. More detail on how data was collected and anonymized should be provided either in “participant recruitment” or in “ethical considerations”. | We have rewritten the Participant Recruitment section in more detail. |
|  | 5. “Pearson’s chi-square and bivariate logistic regression was done to evaluate the independent associations of multiple factors.” This analysis was also performed using excel or any statistical software was used instead? Please modify accordingly. | All the statistical analysis were performed using IBM SPSS version 26. We have rewritten the statistical analyses section to make it clear to all. |
|  | 6. “All the participants who needed RTPCR test were offered the same by Hospital. All the participants with poor IPC practices were recommended for refresher IPC training” this should be moved to in “participant recruitment” instead of being part of “ethical considerations”. | We have shifted these sentences as suggested by the reviewer |
|  | 7. I’m missing information on IRB code approval. It should be stated in the “ethical considerations” section. | Thank you for pointing this out. We have added the IEC code in the current version of manuscript. |
| Results: | 1. Out of a total of 405 HCW approached, 192 were recruited in this study – please add participation rate at the end of the sentence. Results should include information on denominators 192 HCW recruited from how many? Which is the participation? This should be stated clearly in the results section but also in the abstract section. | We want to clarify about enrolment.  Only 405 HCWs had recent exposure to COVID-19 patients and were our potential participants who could be recruited (sample frame). They were screened with the inclusion and exclusion criteria, and 192 were recruited in this study. After excluding 10 participant who were not eligible, 192/395 is the participation rate. |
|  | 2. Please add N= and % for each value to facilitate the comprehension of the manuscript. | We have provided Frequency (N) and percentage (%) of every variable in the tables, but avoided this information in text as it would be duplication of information in the tables. |
|  | 3. “Out of a total of 405 HCW approached, 192 were recruited in this study. All of them were interviewed and blood sampling for serology was done at the baseline visit. Out of them, 139 were also included at end line serology assessment, reason of lost to follow up are highlighted in Figure 2.” This could be moved to methodology rather than a result. This could be part of “participant recruitment” and will be easier to read and understand. | Thank you for your suggestion and we have shifted the information in method section but have revised them in result section for comprehension to the readers |
| Discussion | Discussion should be more elaborated and include an update of the bibliographic references.  50.1% of participants did not provide consent to participate in the study, which is a very low participation rate. This should be stated among the limitations of the study. Also the lost to follow up has to be mentioned in limitations.  Conclusions should be better presented. | Yes this was a limitation, but major reason for this was a lack of time with doctors and nurses, as they were exhausted with their covid duty and were not able to provide time for this study. As suggested we have added these in limitations.  We have redrafted conclusion to make it clearer |
| Tables, figures and supplementary material: | Some principal tables should be move to supplemental, some supplemental should be move to main tables and some tables should be combined.  For example: table 2 should be combined with supplemental table 1 to include all the information, as this is among the main tables of this paper, also, information on p value across different HCW groups should be added.  Supplementary table 2 and 3 should be principal tables as well, and groups should be compared and statistical significances should be added. “We observed a definite trend of adherence to PPE among various health workers as shown in Supplementary Table 2.” How this trend is evaluated? Info should be added.  Some figures need to be eliminated as they do not show relevant information. Others should be improved.  Figure 1: Please review the spelling. The visualization of the figure should be improved. Footnote could be of interest. It could be supplementary material.  Figure 2: Please review the spelling. It could be supplementary material.  Figure 3 could be part of text only as visualization is not the most convenient. Also information regarding statistically significant differences on the text should be considered to be included.  Figure 4 Visualization has to improve, numbers are very hard to read. It could be supplementary material.  Figure 6 could be part of text only; the visualization does not add value to manuscript. Figure 6 information should be combined with table 3. And both should be combined with suppl table 5. Then Suppl table 5 can be a main table of results. | Thankyou for the important suggestions.  We have merdge the table 2 with supplemental table 1, although we could not analyse the association as we lack sufficient samples for each category..  Supplementary table 2 and 3 are labled as principal tables as suggested, although we calculated the statistical significances for Supplementary table 2, but did not have enough sample size for assessing association of individual subpopulation of HCWs for Supplementary table 3.  Figure 1 is made supplementary Figure 1 along with spell check and footnote  Figure 2 is now figure 1 and checked for spelling. We have added more information and now show participants enrolment and retained as principal figure as you have suggested in methods section.  Figure 3 is now figure 2. We have used percentages of the categories for more clarity and calculated significance, as suggested.  Figure 4 is now supplementary figure 2 and we have tried to improve the visualization to improve clarity.  As suggested we have merged the information from figure 6, table 3 and table 5 and prepared new table (Table 6)  The authors have redrafted the whole manuscript. The final version that we are now submitting is after getting edited by professional editing service. |
